# Geographical variation of common childhood illness and its associated factors among under-five children in Ethiopia: spatial and multilevel analysis

**DOI:** 10.1038/s41598-023-27728-8

**Published:** 2023-01-17

**Authors:** Dagmawi Chilot, Mengistie Diress, Yibeltal Yismaw Gela, Deresse Sinamaw, Wudneh Simegn, Amare Agmas Andualem, Abdulwase Mohammed Seid, Desalegn Anmut Bitew, Mohammed Abdu Seid, Habitu Birhan Eshetu, Anteneh Ayelign Kibret, Daniel Gashaneh Belay

**Affiliations:** 1 Center for Innovative Drug Development and Therapeutic Trials for Africa (CDT-Africa), College of Health Sciences, Addis Ababa University, Addis Ababa, Ethiopia; 2 Department of Human Physiology, School of Medicine, College of Medicine and Health Science, University of Gondar, Gondar, Ethiopia; 3 Department of Biomedical Science, Debre Markos University, Debre Markos, Ethiopia; 4 Department of Social and Administrative Pharmacy, College of Medicine and Health Science, University of Gondar, Gondar, Ethiopia; 5 Department of Anesthesia, Wollo University, Dessie, Ethiopia; 6 Department of Clinical Pharmacy, College of Medicine and Health Science, University of Gondar, Gondar, Ethiopia; 7 Department of Reproductive Health, University of Gondar, Gondar, Ethiopia; 8 Unit of Human Physiology, Department of Biomedical Science, College of Health Sciences, Debre Tabor University, Debre Tabor, Ethiopia; 9 Department of Health Education and Behavioral Sciences, University of Gondar, Gondar, Ethiopia; 10 Department of Human Anatomy, School of Medicine, College of Medicine and Health Science, University of Gondar, Gondar, Ethiopia; 11 Department of Epidemiology and Biostatistics, College of Medicine and Health Science, Institute of Public Health, University of Gondar, Gondar, Ethiopia

**Keywords:** Gastrointestinal diseases, Infectious diseases, Respiratory tract diseases, Diseases, Medical research, Risk factors

## Abstract

Although substantial progress has been made in reducing child mortality over the last three decades, the magnitude of the problem remains immense. Ethiopia is one of the countries with a high under-five mortality rate due to childhood illnesses including acute respiratory infections, diarrhea, and fever that varies from place to place. It is vital to have evidence of the factors associated with childhood illnesses and the spatial distribution across the country to prioritize and design targeted interventions. Thus, this study aimed to investigate the spatial cluster distribution and associated factors with common childhood illnesses. Secondary data analysis based on the 2016 Ethiopian Demographic and Health Survey data was carried out. A total weighted sample of 10,417 children was included. The study used ArcGIS and SaTScan software to explore spatial distribution. For associated factors, a multilevel binary logistic regression model was fitted using STATA V.14 software. Adjusted Odds Ratios (AOR) with a 95% Confidence Interval (CI) and *p*-value ≤ 0.05 in the multivariable model were used to declare significant factors associated with the problem. ICC, MOR, PCV, and deviance (−2LLR) were used to check model fitness and model comparison. In this study, the prevalence of common childhood illnesses among under-five children was 22.5% (95% CI: 21.6–23.3%). The spatial analysis depicted that common childhood illnesses have significant spatial variation across Ethiopia. The SaTScan analysis identified significant primary clusters in Tigray and Northern Amhara regions (log-likelihood ratio (LLR) = 60.19, *p* < 0.001). In the multilevel analysis, being rural residence [AOR = 1.39, 95% CI (1.01–1.98)], small child size at birth [AOR = 1.36, 95% CI (1.21–1.55)], high community poverty [AOR = 1.26, 95% CI (1.06–1.52)], mothers aged 35–49 [AOR = 0.81, 95% CI (0.69–0.94)], the household had electricity [AOR = 0.77, 95% CI (0.61–0.98)], the household had a refrigerator [AOR = 0.60, 95% CI (0.42–0.87)], improved drinking water [AOR = 0.82, 95% CI (0.70–0.95)], improved toilet [AOR = 0.72, 95% CI (0.54–0.94)], average child size at birth [AOR = 0.83, 95% CI (0.75–0.94)] were significantly associated with common childhood illnesses. Common childhood illnesses had spatial variations across Ethiopia. Hotspot areas of the problem were found in the Tigray, Northern Amhara, and Northeast SNNPR. Both individual and community-level factors affected common childhood illnesses distribution and prevalence in Ethiopia. Therefore, public health intervention should target the hotspot areas of common childhood illnesses to reduce their incidence in the country.

Despite the world has shown substantial progress in reducing child mortality over the last 30 years, the magnitude remains huge^1–3^. It is a worldwide health priority and one of the Millennium Developmental Goals (MDG) to reduce child mortality^4^. According to the United Nations Children’s Fund (UNICEF) report, about 5.2 million under-five children die in 2019 alone, which indicates on average about 14,000 children died every day^5^. More than half (2.8 million) and almost one-third (1.5 million) of child mortality occurred in sub-Saharan Africa, and Southern and Central Asia, respectively^6^. Countries such as Nigeria, India, Pakistan, the Democratic Republic of the Congo, and Ethiopia accounted for almost half (49%) of all under-five deaths in 2019^7^.

The goal of ending preventable childhood mortality and morbidity is hindered by significant inequalities among countries. Children continue to face widespread regional disparities in their chances of survival. Special attention should be given to the SSA region, where child mortality rates are highest^8,9^. Studies have shown that community and household-level factors have the highest impact on child mortality and morbidity due to Acute respiratory infection (ARIs), diarrhea, and fever^10^. Moreover, low socioeconomic status such as low family wealth index and community poverty, as well as low educational attainment of the child's parents, were consistently reported factors for the high prevalence^11–14^. This requires a strong health delivery system and meaningful child survival interventions to accelerate the pace of child mortality decline.

Ethiopia is among the five countries with a high under-five child mortality rate with an annual rate reduction of 4.7%^7^. In 2019, the country has recorded an average under-five mortality rate of 51 deaths per 1000 live births^15^. Diseases including ARI, fever, and diarrhea are among the major causes of under-five mortality in Ethiopia^16,17^. However, the prevalence has significant variation across the country and is highly concentrated in rural areas, poor, and communities^18–20^. Thus, identifying geographical areas with a high rate of common childhood illnesses using geographical information systems (GIS) and spatial scan statistical analysis (SaTScan) has tremendous importance to guide targeted public health interventions.

Most information regarding morbidity and mortality due to childhood illnesses was derived from health facilities, although many children do not seek medical attention in Ethiopia. In addition, studies have been focused on individual illnesses' prevalence and their factors using standard logistic regression models despite the hierarchical structure of the EDHS data^20,21^. The findings of these studies could be biased estimates since the data were nested within clusters. They may not be representative for estimating the burden of childhood diseases and couldn’t provide a panoramic view of the problem and its associated community-level factors. Given these, the purpose of our study was to investigate the spatial distribution and the associated factors with common childhood illnesses among children younger than five years of age, by using spatial and multi-level analysis.

## Methods and materials

### Study design, setting, and period

The Demographic and Health Surveys (DHS) used a cross-sectional survey study design to collect the data. Secondary data analysis was done based on the fourth survey (EDHS 2016) data. Ethiopia, the 2nd most populous country is situated in the Horn of Africa and most of its population (84%) live in rural areas. It has a total area of 1,100,000 km2 and lies between latitudes 3° and 15°N, and longitudes 33° and 48°E. It has nine regional states (Afar, Amhara, Benishangul-Gumuz, Gambela, Harari, Oromia, Somali, Southern Nations, Nationalities, and People’s Region (SNNPR), and Tigray) and two administrative cities (Addis Ababa and Dire-Dawa).

### Data source and measurements

The data for this study were drawn from recent nationally representative DHS data conducted in Ethiopia. The DHS surveys are routinely collected every five years across low- and middle-income countries using structured, pretested, and validated tools. The DHS survey employs a stratified two-stage sampling technique in each country. In the first stage, Enumeration Areas (EAs) were randomly selected while in the second stage households were selected by systematic sampling. The detailed sampling procedure has been presented in the full EDHS 2016 report^22^. We used the Kids record dataset (KR) file and we included only children under age 5 with at least one of the three diseases (ARI, diarrhea, fever) at any time in the 2 weeks preceding each survey. Therefore, the total weighted sample size analyzed in this study was 10,417.

### Definition of variables

#### Outcome variable

Common childhood illnesses among under-five children were the outcome variable. In this study, the child had an illness when he/she encountered at least one of the three childhood illnesses (ARI, diarrhea, fever), and was categorized as “Yes” while, those who had none of them were categorized as “No”. For the ith child, the dependent variable was represented by a random variable Yi, with two possible values coded as 1 and 0. Therefore, Yi = 1 if the child had at least one of the illnesses (ARI, diarrhea, fever) while Yi = 0 if the child had none of the three illnesses.

#### Independent variables

Major explanatory variables were considered on two levels. Individual-level variables included maternal and child characteristics as well as household characteristics. Whereas, place of residence (urban, rural), community poverty level (low, high), community literacy level (low, high), and community media exposure (low, high) were considered as the community-level factors. To generate community-level variables (community media exposure, community poverty, and community women's education) we did an aggregation of individual-level variables at the cluster level and categorized them^23^ as higher or lower based on a median value.

### Data analysis procedures

#### Spatial analysis

ArcGIS V.10.7 software and SaTScan V.9.6 software were used for exploring spatial distribution, global spatial autocorrelation, spatial interpolation, spatial windows, and for identifying significant hotspot areas of common childhood illnesses. Incremental spatial autocorrelation was done to obtain the maximum peak (clustering) distance where extreme spatial autocorrelation occurs and this was used as a distance band for hotspot analysis. We used 10 distance bands with a starting distance of 121,814 m.

Spatial autocorrelation (Global Moran’s I) was carried out to measure whether common childhood illness patterns were dispersed, clustered, or randomly distributed in Ethiopia^23,24^. Therefore, Moran’s I value close to − 1 indicates the spatial distribution of common childhood illnesses was dispersed, whereas Moran’s I value close to + 1 indicates the spatial distribution of common childhood illnesses was clustered, and an I value of 0 means common childhood illnesses were distributed randomly. For this study, the null hypothesis was common childhood illnesses are randomly distributed. A statistically significant Moran’s I (*p* < 0.05) leads to the rejection of the null hypothesis indicating the presence of significant spatial autocorrelation.

Getis-Ord Gi* statistics hotspot analysis^25^ was used to show significant hotspot areas for common childhood illnesses among under-five children. The spatial interpolation technique was used to predict common childhood illnesses in unsampled areas based on sampled EA measurements. The ordinary Kriging spatial interpolation method, which has the smallest root mean square error value and residuals, was chosen for this study to predict common childhood illnesses in unobserved areas^26^. Spatial scan statistical analysis (SaTScan) using the Bernoulli distribution was used to test for the presence of statistically significant spatial clusters of common childhood illnesses using Kulldorff’s SaTScan V.9.6 software^27^.

### Multi-level analysis

A multilevel binary logistic regression model was fitted to identify significantly associated factors. Variables were extracted from each of the KR files and STATA version 14.2 was used to clean, recode and analyze the data. Four models were applied, comprising the null model (model 0) with no explanatory variables, Model I with individual-level variables, Model II with community-level factors, and Model III with both individual and community-level variables.

Because the models were nested, we used deviance (−2LLR) for model comparison. Accordingly, the model with the lowest Deviance was selected which is Model III. The intra-cluster Correlation Coefficient (ICC) was used to quantify the degree of heterogeneity of common childhood illnesses between clusters. In addition, the Likelihood Ratio test (LR), Proportional Change in Variance (PCV), and Median Odds Ratio (MOR) were computed to measure the variation between clusters. Both community and individual-level variables with a *p*-value ≤ 0.2 in the bi-variable analysis were included in the multivariable model. Adjusted OR (AOR) with 95% CI and *p* < 0.05 were applied to determine significantly associated factors. We used the variance inflation factor (VIF) test to check multicollinearity.

All methods in our study were carried out in accordance with DHS guidelines and regulations. Permission for the dataset was obtained from DHS online request. Informed consent from participants and ethical approval wasn’t a requirement for the ethics committee as this was secondary data.

## Results

### Socio-demographic and economic characteristics of respondents

A total of 10,417 under-five children in Ethiopia were included in this study. About 63.83% of respondents were mothers who had no formal education and more than half (52.13%) of them were mothers in the age group of 25–34 years. The majority of respondents (80.94%) were rural residents with no electricity (77.28%), no refrigerator (93.62%), and substandard floor material (87.74%). With regard to wealth index and place of delivery, about 5376 (53.73%) and 6781 (67.77%) were poor and give birth at home respectively (Table 1).

**Table 1:** Socio-demographic and economic characteristics of respondents in Ethiopia, 2016.

Variables	Categories	Unweighted frequency (%)	Weighted frequency (%)
Age of mothers	15–24	2414 (24.13)	2319 (22.27)
	25–34	5216 (52.13)	5541 (53.19)
	35 +	2376 (23.75)	2557 (24.55)
Mothers educational level	No education	6387 (63.83)	6857 (65.83)
	Primary education	2538 (25.36)	2806 (26.94)
	Secondary and above	1081 (10.80)	753 (7.23)
Mothers marital status	Married	9321 (93.15)	9781 (93.89)
	Not married	685 (6.85)	636 (6.11)
Wealth index	Poor	5376 (53.73)	4885 (46.89)
	Middle	1386 (13.85)	2159 (20.72)
	Rich	3244 (32.42)	3373 (32.38)
Media access	No	6711 (67.07)	7005 (67.25)
	Yes	3295 (32.93)	3412 (32.75)
Household had electricity	No	7733 (77.28)	8802 (84.49)
	Yes	2273 (22.72)	1615 (15.51)
Household had refrigerator	No	9368 (93.62)	10,083 (96.80)
	Yes	638 (6.38)	333 (3.20)
Source of drinking water	Unimproved	3912 (39.10)	4502 (43.22)
	Improved	6094 (60.90)	5915 (56.78)
Type of toilet facility	Unimproved	8155 (81.50)	9217 (88.48)
	Improved	1851 (18.50)	1199 (11.52)
Floor material	Standard	1227 (12.26)	695 (6.68)
	Sub-standard	8779 (87.74)	9721 (93.32)
Child size at birth	Large	3021 (30.19)	3281 (31.49)
	Average	4297 (42.94)	4442 (42.65)
	Small	2688 (26.86)	2694 (25.86)
Place of delivery	Home	6781 (67.77)	7655 (73.49)
	Health facilities	3225 (32.23)	2761 (26.51)
Residence	Urban	1907 (19.06)	1163 (11.16)
	Rural	8099 (80.94)	9254 (88.84)
Community-level women's education	Low	5018 (50.15)	5262 (50.52)
	High	4988 (49.85)	5155 (49.48)
Community poverty	Low	5020 (50.17)	6504 (62.44)
	High	4986 (49.83)	3913 (37.56)
Community-level media usage	Low	4991 (49.88)	4650 (44.64)
	High	5015 (50.12)	5766 (55.36)

### Spatial analysis

#### Spatial and incremental autocorrelation of common childhood illnesses

**Figure 1:**
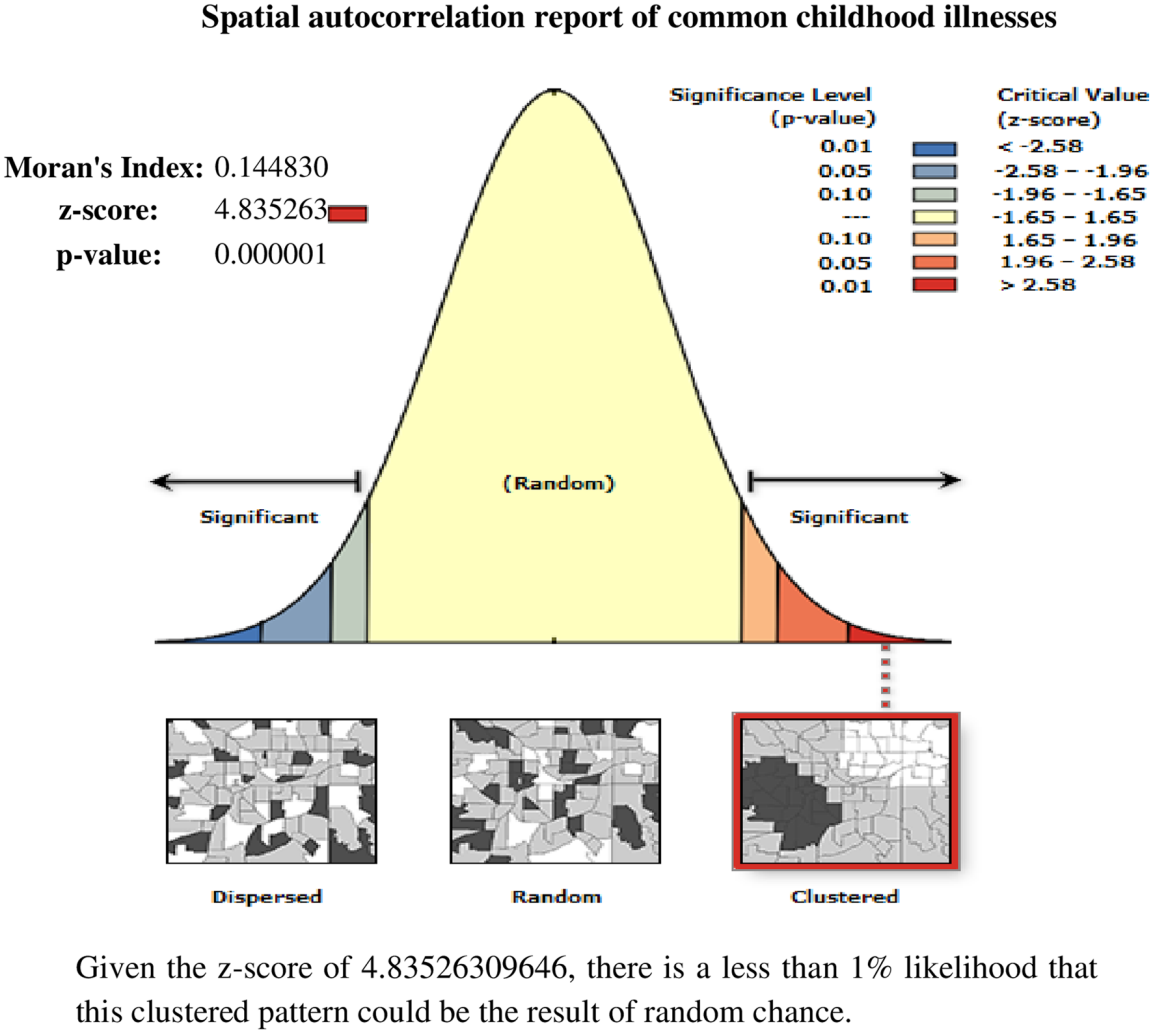
Spatial autocorrelation of common childhood illnesses among under-five children in Ethiopia, 2016 EDHS.

**Figure 2:**
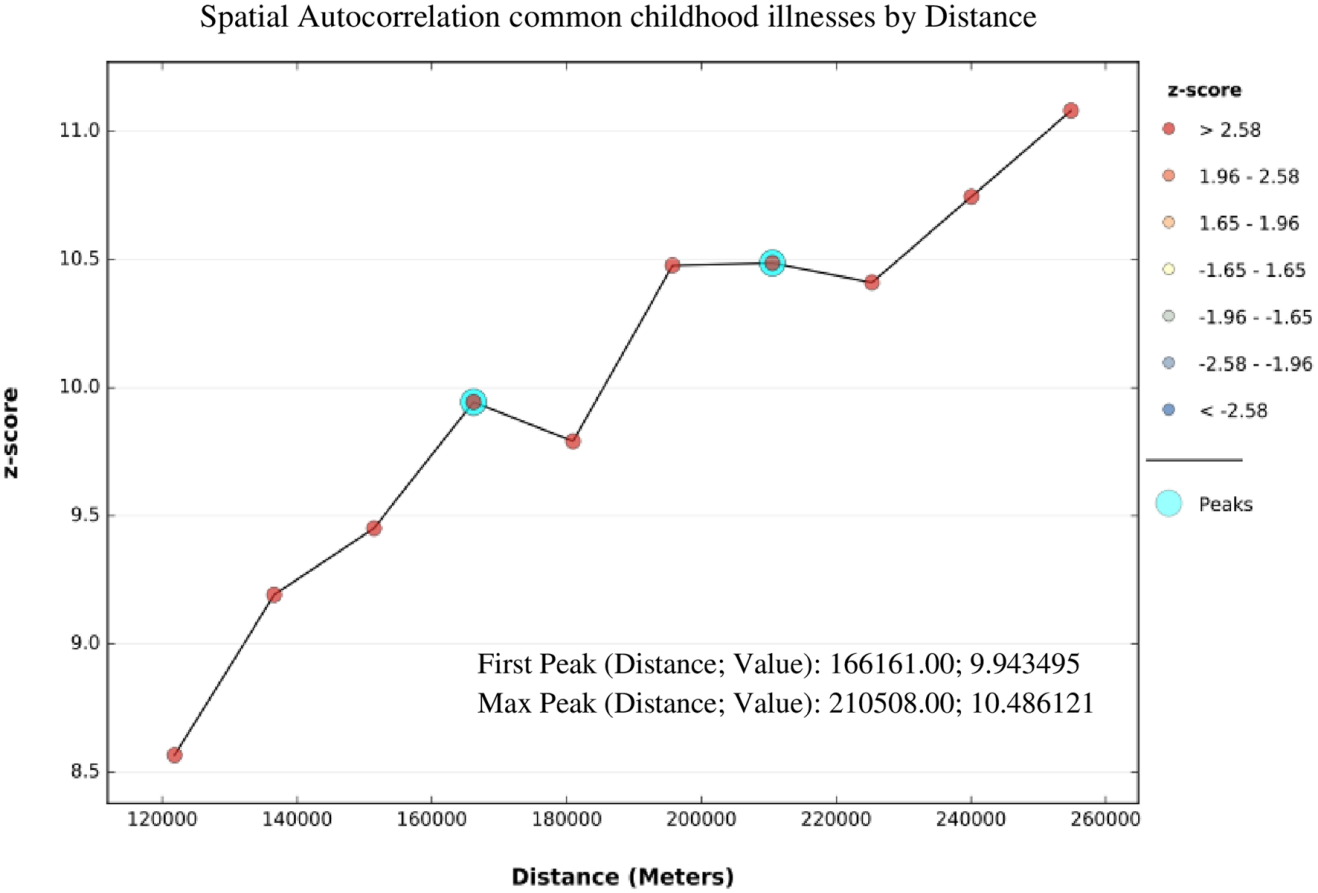
Incremental autocorrelation of common childhood illnesses among under-five children in Ethiopia, 2016 EDHS.

Based on the 2016 EDHS, the spatial distribution of common childhood illnesses significantly varied across Ethiopia, with Global Moran’s I value of 0.15 (*p* < 0.0001). A z-score of 4.83 indicated that there is less than a 1% likelihood that this clustered pattern could be the result of random chance (Fig. 1). The result of incremental autocorrelation revealed significant Z scores, where the first peak was at 166,161 m and a maximum peak was observed at 210,508 m (Fig. 2).

### Spatial distribution and Hotspot analysis of common childhood illnesses

**Figure 3:**
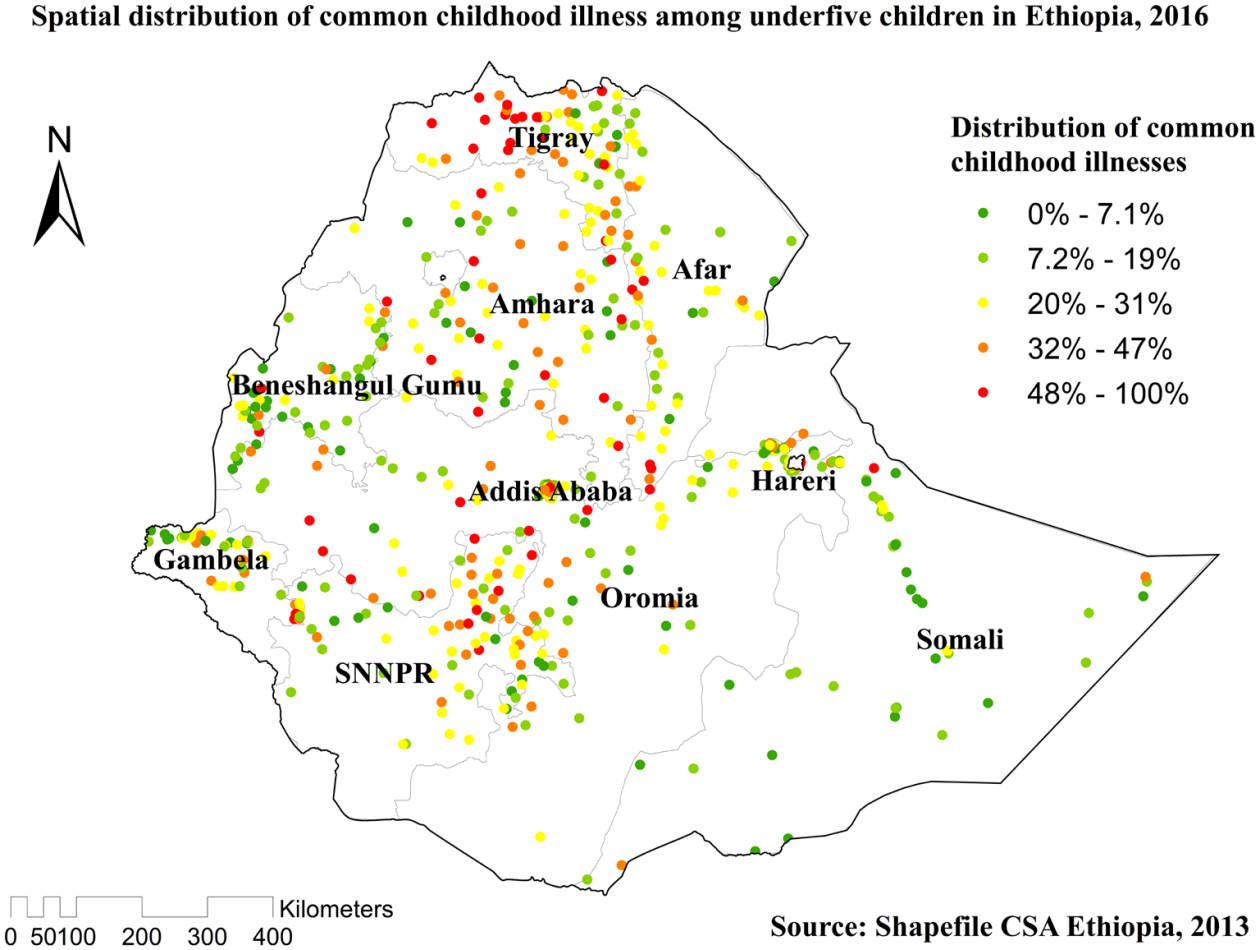
Spatial distribution of common childhood illnesses among under-five children in Ethiopia, 2016 EDHS.

**Figure 4:**
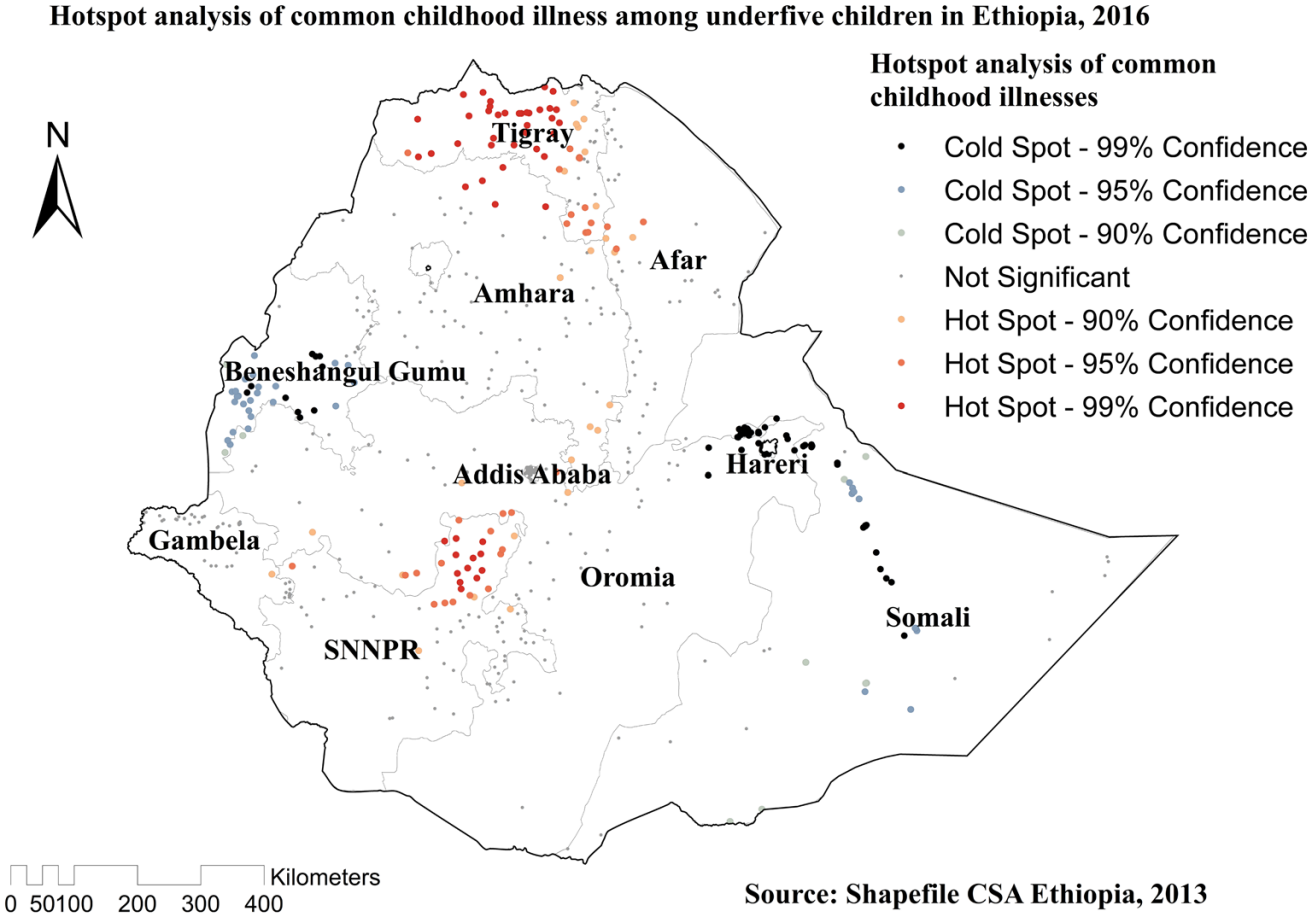
Hotspot analysis of common childhood illnesses among under-five children in Ethiopia, 2016 EDHS.

Significant spatial variation was found in common childhood illnesses in Ethiopia (Fig. 3). The red dots showed the clustering of the proportion of illnesses, whereas the green dots showed a lower proportion of the problem. Hotspot areas of common childhood illnesses were found in Tigray, Northern Amhara, and northern east SNNPR, while cold spot areas of common childhood illnesses were found in West Benishangul-Gumuz, Amhara, Addis Ababa, Oromia region, West Gambella, west Afar, Hareri, Somali and eastern and western SNNPR regions (Fig. 4).

### Spatial SaTScan and Interpolation of common childhood illnesses

**Figure 5:**
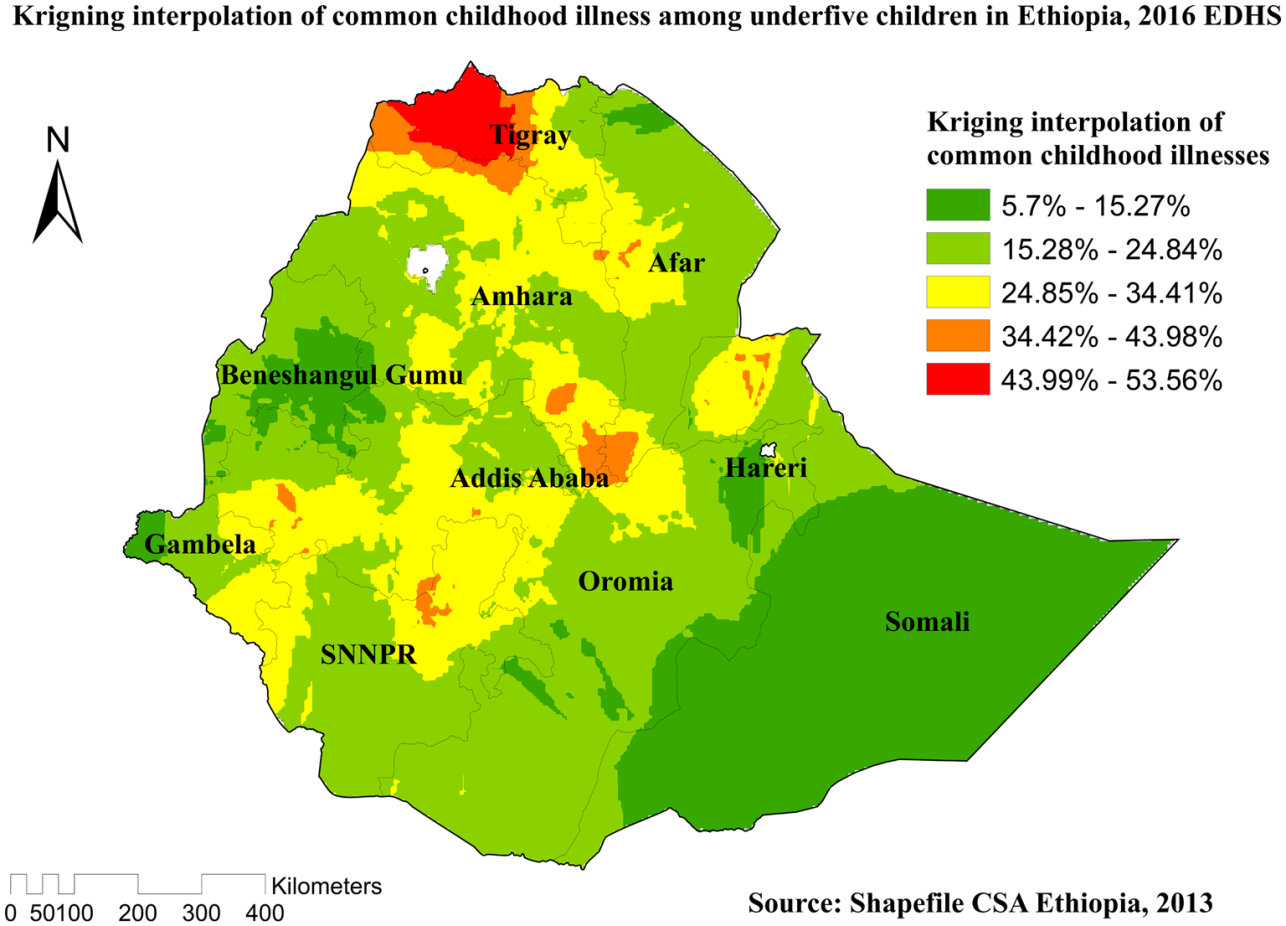
Kringing interpolation of common childhood illnesses among under-five children in Ethiopia, 2016 EDHS.

**Figure 6:**
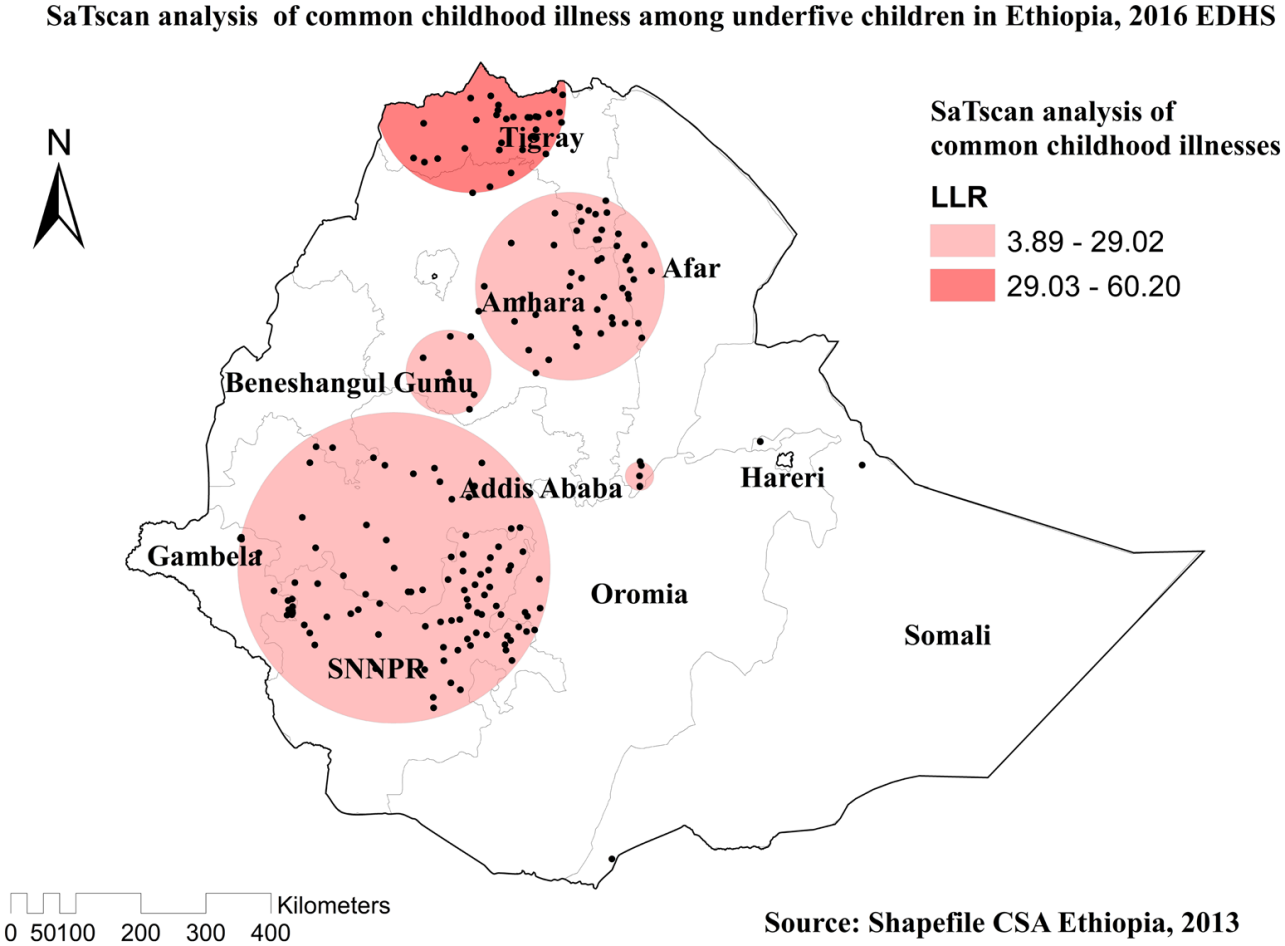
Spatial SaTScan analysis of common childhood illnesses among under-five children in Ethiopia, 2016 EDHS.

Compare to other regions, Western Tigray, South East Amhara, southwest Afar, Northern Somali, West Oromia, and Northwest SNNPR were predicted as the riskiest areas for common childhood illnesses (Fig. 5). In the spatial scan statistical analysis, a total of 135 significant clusters of common childhood illnesses were identified, of which 31 clusters were primary (most likely clusters), located in Tigray and Northern Amhara region centered at 14.390268 N, 37.773392 E of geographical location, with a 146.41 km radius. Children found in the SaTScan window were two times more likely to have common illnesses (RR = 2.04, *P*-value < 0.0001). The red circular ring indicates that the most statistically significant spatial window contains the primary cluster of common childhood illnesses (Table 2 and Fig. 6).

**Table 2:** SaTScan analysis results of common childhood illness in Ethiopia, 2016.

Cluster	Enumeration area (cluster) identified	Coordinate/radius	Population	Case	RR	LLR	*P* value
1* (31)	268, 78, 583, 528, 255, 98, 181, 584, 258, 253, 340, 188, 597, 400, 590, 612, 81, 636, 551, 156, 296, 84, 504, 479, 425, 45, 80, 89, 579, 481, 322	14.390268 N, 37.773392 E / 146.41 km	514	226	2.04	60.19	< 0.0001
2 (104)	432, 486, 62, 447, 227, 76, 489, 586, 411, 154, 502, 142, 207, 306, 338, 577, 174, 113, 331, 177, 126, 262, 41, 555, 272, 477, 360, 565, 223, 118, 141, 537, 271, 388, 280, 359, 297, 234, 373, 119, 53, 470, 294, 23, 180, 162, 558, 434, 437, 554, 325, 204, 406, 399, 299, 420, 20, 243, 459, 376, 46, 552, 168, 465, 371, 161, 526, 347, 14, 485, 633, 609, 197, 326, 139, 505, 54, 217, 148, 304, 408, 70, 450, 448, 517, 216, 308, 578, 215, 503, 391, 349, 522, 86, 12, 634, 589, 365, 114, 147, 469, 63, 47, 291	7.858150 N, 36.733552 E / 239.61 km	1644	495	1.42	29.02	< 0.0001

*Primary clusters.

*LLR* Log-likelihood ratio, *RR* relative risk.

### Multilevel logistic regression analysis of common childhood illnesses in Ethiopia

In the multilevel multivariable logistic regression model; mothers aged 35–49, richest, the household had electricity, the household had a refrigerator, improved toilet facility, improved drinking water, rural residence, child size, high community poverty were statistically associated with common childhood illnesses in Ethiopia (Table 3).

**Table 3:** Multivariable multilevel logistic regression analysis results of both individual-level and community-level factors associated with common childhood illness in Ethiopia, EDHS 2016.

Variables	Categories	Null model	Model I AOR [95% CI]	Model II AOR [95% CI]	Model III AOR [95% CI]
Age of mothers	15–24		1.00		1.00
	25–34		0.92 (0.81–1.04)	**–**	0.91 (0.81**–**1.04)
	35–49		0.81 (0.70–0.94)**	**–**	0.81 (0.69–0.94)**
Mothers educational level	No education		1.00		1.00
	Primary education		1.06 (0.94**–**1.20)	**–**	1.05 (0.92–1.18)
	Secondary& above		0.97 (0.76**–**1.23)	**–**	0.96 (0.76–1.23)
Mothers marital status	Married		1.00	**–**	1.00
	Not married		1.03 (0.85**–**1.26)	**–**	1.04 (0.85–1.27)
Media access	No		1.00	**–**	1.00
	Yes		0.95 (0.83**–**1.01)	**–**	0.94 (0.81–1.08)
Wealth index	Poor		1.00	**–**	1.00
	Middle		0.92 (0.78**–**1.09)	**–**	0.9 (0.76–1.06)
	Rich		0.74 (0.57–0.93)**	**–**	0.71 (0.54–0.91)*
Household had electricity	No		1.00	**–**	1.00
	Yes		0.76 (0.61–0.94)*	**–**	0.77 (0.61–0.98)*
Household had refrigerator	No		1.00	**–**	1.00
	Yes		0.58 (.41–0.83)**	**–**	0.60 (0.42–0.87)**
Source of drinking water	Unimproved		1.00	**–**	1.00
	Improved		0.83 (0.71–0.96)**	**–**	0.82 (0.70–0.95)**
Type of toilet facility	Unimproved		1.00	**–**	1.00
	Improved		0.69 (0.52–0.90)*	**–**	0.72 (0.54–0.94)**
Floor material	Standard		1.00	**–**	1.00
	Sub-standard		0.81 (0.64**–**1.02)	**–**	0.78 (0.62–1.00)
Place of delivery	Home		1.00	**–**	1.00
	Health facilities		0.88 (0.74**–**1.05)	**–**	0.87 (0.73–1.04)
Child size at birth	Large		1.00	**–**	1.00
	Average		0.84 (0.75–0.94)*	**–**	0.83 (0.75–0.94)**
	Small		1.36 (1.20–1.55)***	**–**	1.36 (1.21–1.55)***
**Community-level variables**					
Residence	Urban		**–**	1.00	1.00
	Rural		**–**	1.39 (1.06–1.83)*	1.39 (1.01–1.98)*
Community-level media usage	Low		**–**	1.00	1.00
	High		**–**	1.14 (0.87**–**1.47)	1.07 (0.81–1.40)
Community-level women education	Low		**–**	1.00	1.00
	High		**–**	1.21 (0.98**–**1.50)	1.18 (0.95–1.48)
Community-level poverty	Low		–	1.00	1.00
	High			1.20 (1.02–1.43)	1.26 (1.06–1.52)**
**Random effect**					
	Variance	0.58	0.54	0.51	0.46
	ICC	0.15	0.14	0.13	0.12
	MOR	1.96	1.90	1.88	1.74
	PCV	Reff	7.41	13.72	26.09
**Model comparison**					
	Log likelihood ratio	− 5650	− 5588	− 5641	− 5581
	Deviance	11,300	11,176	11,282	11,162
	Mean VIF		1.51	1.13	1.49

*P-value < 0.05, **P-value < 0.01, ***P-value < 0.001.

*ICC* Inter cluster correlation coefficient, *MOR* Median odds ratio, *PCV* Proportional change in variance,* AOR* Adjusted odds ratio, *CI* Confidence interval,* VIF* Variance inflation factor.

## Discussion

This study examined the spatial distribution, individual, and community-level factors associated with common childhood illnesses in Ethiopia. The spatial distribution of common childhood illnesses significantly varied across the country, with high risk in the Tigray and Northern Amhara regions. Significant hotspot areas of common childhood illnesses were detected in the Tigray, Northern Amhara, and Northeast SNNPR regions. The geographical difference in common childhood illnesses across the regional states might be attributable to the regional disparity of accessibility of health services, shortage of safe and adequate drinking water supply, variation of food consumption, poor household characteristics, and unimproved latrine facilities which would increase the transmission of illnesses^22,28–30^. This study could be helpful that responsible bodies to give priority to regions that were at higher risk of the problem.

In the multilevel analysis, different individual and community factors were significantly associated with common childhood illnesses. This study identified lower odds of common childhood illnesses among children born from women aged 35–49 compared with women aged 15–24. This finding is consistent with a previous study conducted in Tanzania^31^. The possible justification for this could be that aged mothers may report a higher frequency of episodes of fever, diarrhea, and ARI than younger women as a result of experience.

The odds of common childhood illnesses were lower among children from families with high household wealth, and this was consistent with previous findings in low-income to middle-income countries^32–35^. Besides, high community poverty increased the odds of common childhood illnesses in Ethiopia. The possible explanation might be that poverty is strongly associated with food insecurity, living standard, and housing conditions which could greatly affect the well-being of a child. Hence children from households and communities with low wealth status may not have an access to enough food, clean water, improved toilets, clean house, refrigerator, and other basics which could promote the transmission of illnesses.

Children from households who had electricity, a refrigerator, improved drinking water, and improved toilets were less likely to have common childhood illnesses compared to their counterparts. This finding aligned with previous studies conducted elsewhere^36–40^. Improved housing with improved drinking water, improved sanitation, access to electricity, had a refrigerator, may be protective against infectious childhood infectious diseases. Childhood illness is due to exposure to disease-causing organisms; therefore, this perhaps depends on housing conditions and the personal hygiene of the household.

Being a rural resident was associated with higher odds of common childhood illnesses. Our finding was inconsistent with several studies done in Ethiopia^20^, Nigeria^41^, and Tanzania^31^. The possible explanation for this could be that in rural areas of Ethiopia people lack pure water, electricity is accessible to very few households, no improved toilets compared to the urban and generally the living standard is low. Moreover, in rural areas, the prevalence of open defecation is high^42^, and people often use wood, animal dung, straw, and other organic materials as a source of fuel to cook food, which exposes children for illnesses such as diarrhea, ARI and fever. Therefore, children born from rural residents could me more vulnerable for common illnesses.

In this study, child size at birth was significantly associated with common childhood illnesses. Small child size at birth was associated with high odds of the problem, whereas average child size at birth was associated with low odds. Previous studies have also shown small size at birth has a significant impact on child mortality and morbidity^43–46^. This could be justified that small size at birth is an important indicator of the child’s vulnerability to the risk of childhood illnesses and to predict the child’s future health, development, and chances of survival.

This study has several strengths and limitations. Among the strengths of our study included a nationally representative sample of under-five children, hence this result may be generalized to Ethiopian children. Moreover, we have done spatial analysis that allows an understanding of the geographic variation of the problem and multilevel analysis to accommodate the hierarchical nature of the EDHS data in estimating the determinant factors. However, this study had limitations including, since EDHS collects the data in cross-sectional design causality cannot be inferred. In addition, the SaTScan analysis detects only circular clusters, irregularly shaped clusters were not detected.

## Conclusions

Common childhood illnesses had spatial variations across Ethiopia. Hotspot areas of the problem were found in the Tigray, Northern Amhara, and Northeast SNNPR. Both individual and community-level factors affected common childhood illnesses distribution and prevalence in Ethiopia. Therefore, public health intervention should target the hotspot areas of common childhood illnesses to reduce their incidence in the country.

## Data Availability

Data are available online in a public, open-access repository (www.measuredhs.com/data).

## Abbreviations

AOR: Adjusted odds ratio
ARI: Acute respiratory infection
EDHSs: Ethiopian demographic and health surveys
ICC: Intracluster correlation coefficient
KR: Kids record
MDG: Millennium development goal
MOR: Median odds ratio
PCV: A proportional change in variance
SaTScan: Spatial scan statistical analysis
SSA: Sub-Saharan Africa
UNICEF: United Nations International Children's Emergency Fund
WHO: World Health Organization
